# Increased α-Defensins 1-3 Production by Dendritic Cells in HIV-Infected Individuals Is Associated with Slower Disease Progression

**DOI:** 10.1371/journal.pone.0009436

**Published:** 2010-02-25

**Authors:** Marta Rodríguez-García, Núria Climent, Harold Oliva, Víctor Casanova, Rafael Franco, Agathe Leon, José M. Gatell, Felipe García, Teresa Gallart

**Affiliations:** 1 Services of Immunology, Hospital Clínic de Barcelona, Barcelona, Spain; 2 Institut d'Investigacions Biomediques August Pi i Sunyer (IDIBAPS)-AIDS Research Group, and Catalonian Center for HIV Vaccines (HIVACAT), Barcelona, Spain; 3 Hospital Clínic de Barcelona, University of Barcelona School of Medicine, Barcelona, Spain; 4 Institut d'Investigacions Biomèdiques August Pi i Sunyer (IDIBAPS), and Department of Biochemistry and Molecular Biology, Barcelona, Spain; 5 Faculty of Biology, University of Barcelona, Barcelona, Spain; 6 Centro de Investigación Médica Aplicada, University of Navarra, Pamplona, Spain; 7 Infectious Diseases and AIDS Unit, Hospital Clínic de Barcelona, Barcelona, Spain; University of Sao Paulo, Brazil

## Abstract

**Background:**

Defensins are natural endogenous antimicrobial peptides with potent anti-HIV activity and immuno-modulatory effects. We recently demonstrated that immature dendritic cells (DC) produce α-defensins1-3 and that α-defensins1-3 modulate DC generation and maturation. Since DC-HIV interaction plays a critical role during the first steps of HIV infection, we investigated the possible impact of α-defensins1-3 production by DC on disease progression.

**Methodology/Principal Findings:**

Monocyte-derived DC (MDDC) were analyzed comparatively in healthy controls (HC) and HIV-infected patients, including untreated “elite” and “viremic” controllers, untreated viremic non-controllers and antiretroviral-treated patients. We found that production of α-defensins1-3 was significantly increased in MDDC from HIV-infected patients versus HC, and this increase was mainly due to that observed in controllers, while in non-controllers the increase was not statistically significant (controllers vs. HC, p<0.005; controllers vs. non-controllers p<0.05). Secreted α-defensins1-3 by immature MDDC positively correlated with CD4 T cell counts in controllers, but not in non-controllers. Moreover, independently of their clinical classification, HIV-infected patients with higher α-defensins1-3 secretion by immature MDDC showed slower disease progression, measured as no decrease in the number of CD4+ T-cells below 350 cell/mm^3^, lower increase of plasma viral load and no initiation of treatment over time. Plasma alpha-defensins1-3 levels lacked any relationship with immunologic and virologic parameters.

**Conclusions/Significance:**

High production of α-defensins1-3 by immature DCs appears as a host protective factor against progression of HIV-1infection, suggesting potential diagnostic, therapeutic and preventive implications. This protective effect may arise from the activity of α-defensins1-3 to damage the virions prior and/or after their internalization by immature DC, and hence favoring a more efficient viral processing and presentation to HIV-specific CD4+ T cells, without or with a minor rate of transmission of infectious HIV-1 virions.

## Introduction

Defensins are natural endogenous antimicrobial peptides with potent anti-HIV-1 activity [1], [2]. According to structural characteristics, two subfamilies of defensins exist in humans, α- and β-defensins, both with anti-HIV activity[3]. Neutrophils are the main cellular source for α-defensins1-3, which are also designated human neutrophil peptides (HNP1-3)[4], although other leukocyte subsets also produce them[2], [5]–[7]. Apart from their direct anti-HIV-1 effect[5], [8]–[10], α-defensins1-3 display multiple immunostimulatory activities[11], including chemoattraction of naive T cells and immature dendritic cells (imDCs)[12], induction of cytokine and chemokine production[13]–[15] and modulation of the expression of HIV receptors and coreceptors [16].

Recently, we demonstrated that immature (im) monocyte-derived DC (MDDC) from healthy individuals produce α-defensins1-3 [17] and that α-defensins1-3 are able to modulate the maturation and differentiation process of MDDC[18]. MDDC are a valid and widely used *in vitro-*generated model of myeloid DCs [19]–[23].

Myeloid or classical DC are key cells for the generation and regulation of the adaptive immune T-cell responses. They are the most potent professional antigen-presenting cells, unique in their capacity to induce the antigen-specific activation of naïve CD4 and CD8 T cells *in vivo* and *in vitro*, as well as in their ability to induce the response of CD8 T cells via MHC-class I molecules to exogenous non-infectious pathogens, a process called cross-priming or cross-presentation[24]–[28]. Immature myeloid DCs are cells specialized in internalizing exogenous components that reside in the skin, mucosal territories and other non-lymphoid tissues, where they act as sentinels to detect invading pathogens and foreign antigens. Once they engulf a pathogen or an antigen in an inflammatory microenvironment, undergo a process of maturation and migration to proximal lymphoid tissues, where they appear as the interdigitating DC in the T-cell areas, and contact with antigen-specific T cells and induce their activation [24]–[28]. Immature myeloid DC such as Langerhans cells in the mucosal epithelium and interstitial DC in the subepithelial mucosal tissues are thought to be the first cell types that contact with HIV-1, which exploits the biology of DCs to spread the infection to HIV-1-specific and HIV-1-nonspecific CD4 T cells[29]–[31]. Nevertheless, DCs are required for induction of T-cell responses to intracellular pathogens [32], and the capacity of DCs loaded with infectious and non-infectious HIV-1 virions to activate naïve HIV-1-specific CD4 and CD8 T cells has been recently demonstrated *in vitro* [33]. Therefore, the initial contact of HIV-1 with DCs can result in opposite outcomes, either beneficial in inducing strong HIV-specific T-cell responses or deleterious in promoting the spread and dissemination of HIV-1 among HIV-specific and HIV-nonspecific CD4 T-cells, leading to the progressive depletion of CD4 T cells that characterize the natural history of chronic HIV-1 infection. In that setting, given the strong anti-HIV activity of α-defensins1-3, we hypothesized that a high production of α-defensins1-3 by immature DC could contribute to determine a beneficial outcome of the HIV-1-DC interaction that would involve increased and optimal HIV-1-specific T-cell responses, which in turn would retard the progression of the disease. To gain insight about this hypothesis, *in vitro* generated immature and mature MDDC were analyzed for their capacity to produce α-defensins 1-3 comparatively in healthy controls (HC) and HIV-infected patients, including untreated “elite” and “viremic” controllers, untreated viremic non-controllers and antiretroviral-treated patients.

## Materials and Methods

### Study Subjects

#### 1. Ethics statement

Patients and control subjects were recruited at the Hospital Clinic Universitari, Barcelona, after an informed consent, following the approved rules of the Clinical Research and Ethics Committee of our Institution. **2. Characteristics.** The study included healthy, non-infected controls (HC) and HIV-1-infected subjects. The HIV-1-infected individuals were classified as: elite controllers, with plasma viral loads (PVLs) below 50 RNA copies/ml in all the determinations in the absence of therapy; viremic controllers, with PVLs higher than 50 and lower than 5000 RNA copies/ml without therapy[34]; viremic non-controllers, patients with PVLs higher than 5000 RNA copies/ml without therapy; patients with antiretroviral therapy. All patients had CD4 T cell counts higher than 450 cell/mm^3^. The characteristics of the patients are shown in table 1.

**Table 1:** Characteristics of the patients included in the study.

Characteristic	ELITE (n = 4)	VC (n = 15)	VNC (n = 11)	HAART (n = 9)
Sex (male/female)	3/1	9/6	11/0	9/0
Geographic origin (Europe/Other)	3/1	12/3	9/2	8/1
Age Mean (min-max)	47.5 (41–63)	37.6 (26–54)	36.2 (28–59)	41.1 (26–56)
CD4 T cell counts (cell/micr)	803±275.6	741±114	595±170	780±225
HIV load (log copies/ml)	1.69±0	3.11±0.41	4.23±0.36	2.41±1.11

### Generation of Monocyte-Derived Dendritic Cells (MDDC)

MDDCs were generated from human monocytes as previously reported[15], [17], [35], [36] from volunteer healthy donors or HIV-1-infected patients. Briefly, peripheral blood mononuclear cells (PBMC) were isolated immediately after venous blood extraction by standard Ficoll density gradient centrifugation, and then incubated in cell culture dishes for 2 h at 37°C, resuspended at a concentration of 3 million PBMC per ml in serum-free X-VIVO15 culture medium (BioWhittaker, Cambrex) supplemented with 1% of AB human serum. Adherent cells (≥95% CD14+) were washed (3x) in pre-warmed (at 37°C) serum-free X-VIVO10 medium (BioWhittaker, Cambrex), and then differentiated to immature MDDC in a cell culture for 5 days in a total volume of 6 ml of complete DC medium, consisting of serum-free X-VIVO15(BioWhittaker, Cambrex), containing a final concentration of 1,000 units/ml IL-4 (Strathmann Biotec AG, Hamburg, Germany) and 1,000 units/ml of GM-CSF (Peprotech, London, UK). IL-4 and GM-CSF at the same indicated concentration were added at days 0, 3 and 5. Complete XVIVO-15 medium was also supplemented with 1% human AB serum, 50 µg/ml gentamycin (Braun B.) and 2.5 µg/ml fungizone (Bristol-Myers Squibb). To obtain mature MDDC, a cocktail of proinflammatory cytokines [17], [18], containing IL-1β (10 ng/ml), IL-6 (1000 U/ml) and TNF-α (1000 U/ml) (Strathmann Biotec AG) was added on day 5, and the cell culture prolonged for two days. The purity and immunophenotype of immature and mature MDDC was assessed by flow cytometry analysis using commercially labeled monoclonal antibodies (mAbs) against surface markers (See below, “Flow Cytometry”).

### Real Time RT-PCR

The expression of α-defensins1-3 mRNA was evaluated by real time RT-PCR in immature and mature MDDC as previously described[17]. Total RNA was extracted with Trizol Reagent (Invitrogen Corporation, Paisley, Scotland), and cDNA generated as reported. The cDNAs were amplified using LightCycler FastStart DNA Master^PLUS^ SYBR Green I kit (Roche, Penzberg, Germany). Real-time PCR was carried out for 45 cycles using the LightCycler instrument (Roche) and the specific primers (Sigma-Aldrich) described previously[17]. PBMC were used as a positive control[37]. To calculate relative levels of α-defensins1-3 mRNA, β-2-microglobulin mRNA levels were used as an endogenous control[36], [38] to normalize mRNA quantities. Relative mRNA levels were calculated using this formula[39]: Relative mRNA expression  = 2 ^– (Ct of α-defesins 1-3 – Ct of endogenous control)^×10^3^. All cDNA samples were amplified in duplicates.

### ELISA

The levels of α-defensins1-3 in cell-free culture supernatants and plasmas were quantified using the commercial HNP 1-3 ELISA test Kit (Hycult biotechnology) following the manufacturer instructions. The supernatants of immature MDDCs were collected on day 5 of the cell culture of monocytes in the presence of IL-4 +GM-CSF, before adding the maturation cytokine cocktail. The supernatants of mature MDDC were collected two days after the addition of the maturation cocktail.

### Flow Cytometry

The immunophenotype of immature and mature MDDCs was assessed by two-color flow cytometry using FITC- and PE- conjugated mAbs against CD14, CD80, CD83, CD86, CD11c, CD40 and HLA-DR (BD Biosciences). Also, CD3, CD19 and CD56 were used to assess the purity of the cell cultures, resulting to be lower than 2% of the total population. FITC- and PE-conjugated isotype-matched monoclonal antibodies of unknown specificity were used as negative controls.

Intracellular staining of α-defensins 1-3 was performed as described before with minor modifications [17]. Briefly, immature MDDCs were pretreated with monensin (Golgi Stop, BD Biosciences) for 8 h and surface stained with FITC-conjugated CD40 (BD Biosciences). MDDCs were then fixed and permeabilized with the Cytofix-Cytoperm Plus kit (BD Biosciences) for 20 min, incubated with biotinylated mouse mAb anti-human α-defensins 1-3 (Clone D21, Hycult Biotechnology, BD Bioscience) for 30 min and then with PE-conjugated streptavidin (1/1000), BD Biosciences) during 30 min. The negative control was FITC-conjugated isotype control (BD Biosciences) and biotin-conjugated mouse IgG1 isotype control (Southern Biotechnology, Birmingham, AL, USA).

The cells were analyzed in a FACScan flow cytometer (Beckton Dickinson). Data obtained were analyzed with the FlowJo software (Tree Star, Inc. Ashland, OR). Expression of surface and intracellular markers was measured by the percentage of positive cells and the geometric mean fluorescence intensity.

### Statistical Analysis

Data were analyzed using the GraphPad Prism 5.0 software. The non-parametric tests U-Mann Whitney for the comparison of two groups or Kruskal-Wallis followed by Dunns post test for the comparison of more than two groups were used. For the analysis of the correlation the non-parametric test of Spearman was applied. The follow-up of patients for the analysis of mortality curves ended at the month when the event occurred or on April 15, 2009, whichever occurred first. The differences between the mortality curves were analyzed with the log-rank test. For all the tests used, a two sided P value <0.05 was considered statistically significant.

## Results

### Increased α-Defensins1-3 Production by MDDC from HIV-Infected Subjects

To define the levels of α-defensins1-3 produced by immature and mature MDDC from HIV-negative healthy controls (HC) and HIV-1-infected patients, we first performed a comparative analysis of α-defensins1-3 secretion and mRNA relative expression between HC (n = 15) and HIV-infected patients (n = 39). Secreted levels in the culture supernatants were assessed by ELISA and mRNA expression by real time RT-PCR.

**Figure 1:**
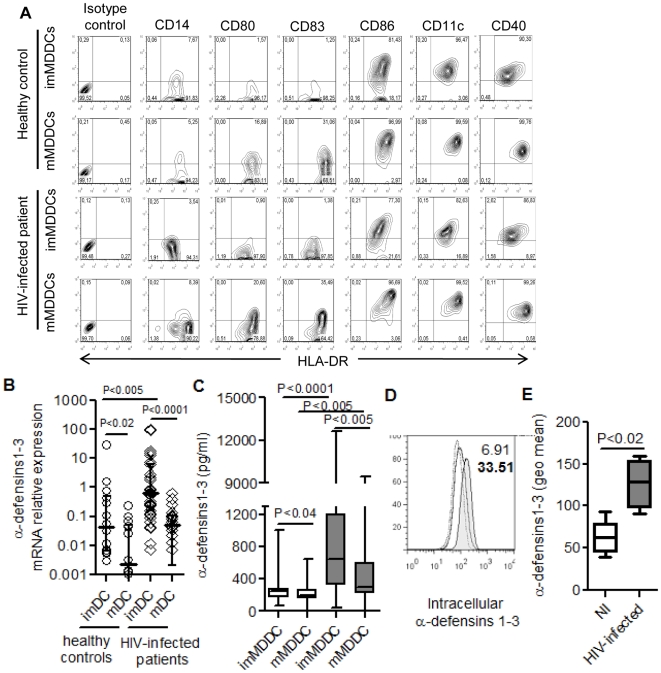
Production of α-defensins 1-3 by immature (im) and mature (m) MDDCs. (A) Immature and mature MDDC immunophenotype from one representative healthy control individual and one HIV-infected subject. X-axis represents HLA-DR expression and Y-axis represents the surface markers indicated. The percentage of positive cells are shown in each quadrant. (B) Relative expression of α-defensins 1-3 mRNA by real time RT-PCR in healthy control individuals (n = 15; open circles) and HIV-infected subjects (n = 39; open rhombus). Dots represent each patient and horizontal lines represent median ± interquartile ranges. Undetectable samples from mMMDC in HC and HIV-infected patients are not represented in the figure. (C) Levels of α-defensins 1-3 detected in MDDC culture supernatants from healthy non-infected controls (n = 15; white boxes) and HIV-infected subjects (n = 39; grey boxes). Boxes represent interquartile ranges, horizontal lines inside each box indicate the median and whiskers indicate maximum and minimum values. (D) Intracellular staining and flow cytometry analysis of α-defensins 1-3 in immature MDDCs. MDDCs were treated with monensin for 8h and double-stained for CD40 and α-defensins 1-3. CD40 positive cells were selected and the histogram corresponds to positive cells for intracellular α-defensins 1-3. Histogram represents the isotype control (dots line), healthy control (open line) and HIV-infected subject (filled histogram). Numbers indicate the percent of positive cells for healthy control (normal number) or HIV-infected patient (bold numbers). Representative result of four different healthy controls and four HIV-infected patients. (E) Geometric mean fluorescence intensity for intracellular α-defensins 1-3 in non-infected individuals (n = 4; white box) and HIV-infected subjects (n = 4; grey box). Boxes represent interquartile ranges, horizontal lines inside each box indicate the median and whiskers indicate maximum and minimum values.

The immunophenotype of immature and mature MDDC was assessed in all individuals. Fig. 1A shows a typical representative flow cytometry analysis from a HC subject and an HIV-1-infected patient. As expected from our previous studies[17], [18], [36], [40] with MDDC from HC, generated in the same conditions as in the current study, after 5 days of cell culture in the presence of GM-CSF and IL-4, monocytes had differentiated into immature MDDCs (imMDDC); these were mostly CD14-negative (≥92%) and virtually all positive (≥98%) for HLA-DR and also mostly positive for CD40, CD11c and CD86, while they lacked expression of CD80 and CD83, markers of mature DC immunophenotype. Maturation was induced by two additional days of culture in the presence of the maturation-cytokine cocktail (Il-1β+IL-6+TNF-α). In accord with our previous studies mentioned above[17], [18], [36], [40], the immunophenotype of mature MDDC (mMDDC) was characterized by an increase in the expression intensity of HLA-DR, CD40, CD86 and CD11c, and in the expression of CD80 and CD83 in a notable proportion of cells. The up-regulation of these markers is a typical feature of DC maturation and was observed in all the individuals included in the study.

As shown in Fig. 1B and 1C, we found that imMDDCs from HIV-negative HC produced higher levels of α-defensins1-3 than mature mMDDCs, in terms of both mRNA relative expression (Fig. 1B; p<0.02) and levels secreted in the supernatants (Fig. 1C; p<0.04). In most cases, α-defensins1-3 mRNA levels in mMDDCs from HC were undetectable or barely detectable. These findings in HC confirm our previous results with a smaller group of healthy individuals [17]. In HIV-infected patients, the production of α-defensins1-3 by imMDDC was also higher than in mMDDC as measured by both mRNA relative expression (Fig. 1B; p<0.0001) and secreted levels in the supernatants (Fig. 1C; p<0.005). The comparison between HC and HIV-infected patients showed that the production of α-defensins1-3 was significantly higher in HIV-infected patients than in HC subjects as assessed by both mRNA expression (fig. 1B; p<0.005) and secreted levels in the supernatants (fig. 1C; p<0.0001). Among HIV-1-infected patients, there was a higher number of individuals with detectable levels of mRNA in mMDDCs compared to HCs, but these levels were clearly lower than those found in imMDDCs (Fig. 1B, p<0.0001).

Additionally, to confirm that the α-defensins1-3 secreted in the supernatants were constitutively produced by imMDDCs, intracellular expression of α-defensins1-3 was analyzed by flow cytometry (Fig. 1D). Immature MDDCs from HCs and HIV-infected patients were treated with monesin for 8 h to accumulate the α-defensins1-3 inside the cell. Flow cytometry analysis after monensin treatment revealed that 5.3±2.1% of imMDDC from HCs contained intracellular α-defensins1-3, an expected finding given our previous study with healthy individuals [17]. In imMDDC from HIV-infected patients, the analysis of the intracellular expression of α-defensins1-3 showed a significant increase in the percentage of positive cells (24±4.8%; figure 1D) as well as in the geometric mean intensity compared to HCs (P<0.02; figure 1E). This higher expression of intracellular α-defensins1-3 in imMDDC of HIV-infected patients compared to HC is in agreement with the higher levels of RNA expression and α-defensins1-3 secretion.

Since in both HIV-infected patients and HC subjects, imMDDC were the main α-defensins1-3-producing cells as compared to mMDDC, all the following analyses were done using imMDDC.

### Increased α-Defensins1-3 Production by imMDDC in Controller versus Non-Controller HIV-Infected Patients

Given the increased production of α-defensins1-3 in the HIV-infected group, we next analyzed comparatively the different subgroups of HIV-infected subjects. These subgroups included (i) “elite controllers” with PVL persistently <50 RNA copies/ml; (ii) “viremic controllers” with PVL between 50 and 5000 RNA copies/ml; (iii) untreated chronic HIV-infected viremic subjects with PVL >5000 RNA copies/ml; and (iv) chronic HIV-infected patients receiving HAART.

**Figure 2:**
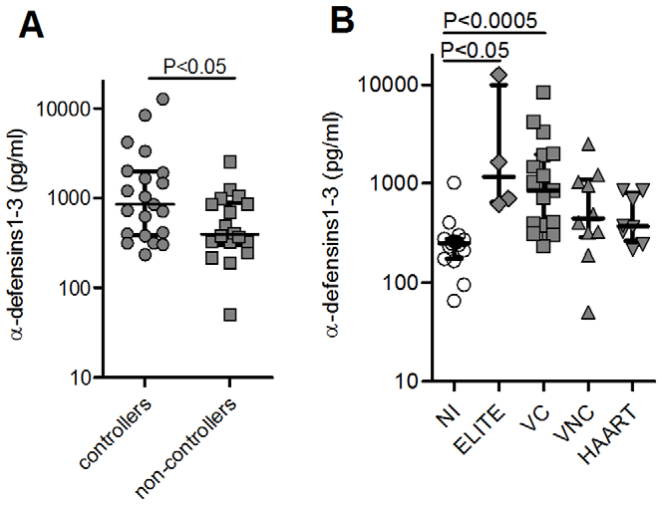
Levels of α-defensins 1-3 produced by imMDDC from HIV-infected individuals. (A) Comparison between the secreted levels of α-defensins 1-3 by imMDDC from HIV-controllers (elite controllers and viremic controllers; n = 19) and HIV non-controllers (viremic non-controllers and patients with HAART; n = 20). (B) Secreted levels of α-defensins 1-3 by imMDDC from healthy non-infected (NI; n = 15), elite controllers (ELITE; n = 4), viremic controllers (VC; n = 15), viremic non-controllers (VNC; n = 11) and patients receiving HAART (HAART; n = 9). Dots indicate each patient and lines represent median ± interquartile ranges.

The comparison between controllers (elite + “viremic” controllers) and non-controllers (untreated viremic + treated patients) showed a significantly higher secretion of α-defensins1-3 by imMDDC from those individuals able to spontaneously control the infection than in subjects with no control of the infection (fig. 2A; p<0.05). We also found that the secretion of α-defensins1-3 in the two controller groups was significantly higher than in HC subjects. In the non-controller groups this secretion remained slightly increased compared to HC but without statistical significance (Fig. 2B).

These data collectively indicate that α-defensins1-3 secretion by imMDDC is clearly increased in HIV-infected individuals who spontaneously control the infection.

### Secreted Levels of α-Defensins1-3 by imMDDC Correlated with CD4 T Cell Counts but Not with PVL

We next examined the possible relationship between α-defensins1-3 secretion by imMDDC and two markers of disease progression: CD4 T cell count and PVL. For this comparison we employed CD4 T cell counts and PVL found on the same date of blood extraction for generation of imMDDC.

**Figure 3:**
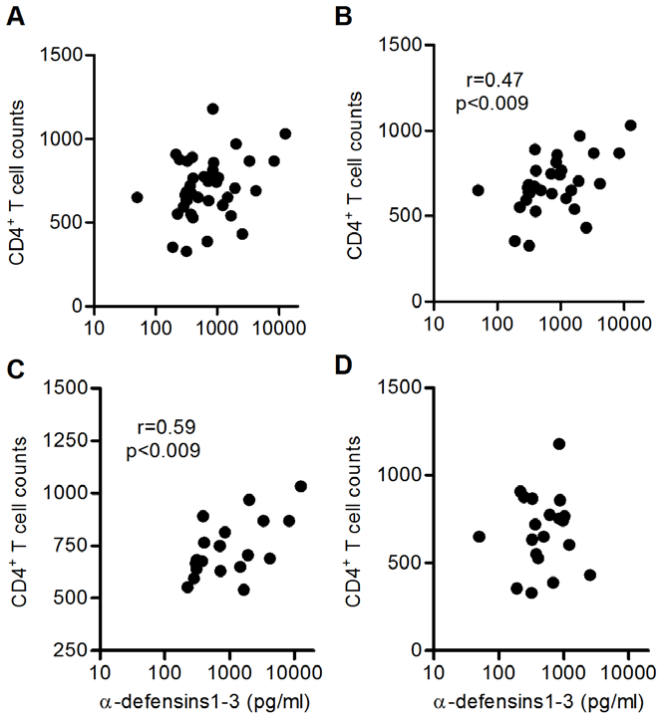
Positive correlation between secreted levels of α-defensins 1-3 by imMDDC and CD4 T cell counts. (A) HIV-infected patients (n = 39), (B) patients not receiving HAART (n = 30), (C) controllers (elite and viremic controllers; n = 19), and (D) non-controllers (viremic non-controllers and patients with HAART; n = 20). Spearman correlation test was applied.

In the whole cohort of HIV-infected patients, secreted α-defensins1-3 by imMDDC did not correlate with CD4 T cell counts (Fig. 3A). However, a positive correlation was found when the patients with HAART were eliminated from the analysis (Fig. 3B; Spearman r = 0.47; p<0.009). In dividing the whole cohort between controllers and non-controllers, the correlation was only maintained in controllers (Fig. 3C; Spearman r = 0.58; p<0.009), but not in non-controllers (Fig. 3D).

**Figure 4:**
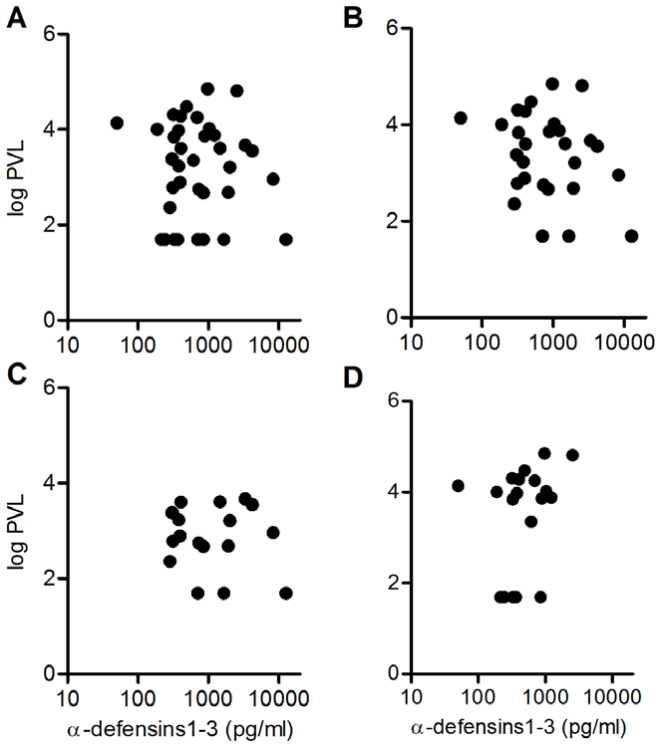
No correlation between secreted levels of α-defensins 1-3 by imMDDC and plasmatic viral load (PVL). (A) HIV-infected patients (n = 39), (B) patients not receiving HAART (n = 30), (C) controllers (elite and viremic controllers; n = 19), and (D) non-controllers (viremic non-controllers and patients with HAART; n = 20). Spearman correlation test was applied.

No correlation was found between PVL and levels of α-defensins1-3 secreted by imMDDC (Fig. 4A), even eliminating untreated patients from the analysis (fig. 4B) or separating controllers (Fig. 4C) from non-controllers (Fig. 4D).

### Plasma Levels of α-Defensins1-3 Showed No Differences between Controllers and Non-Controllers HIV-Infected Patients

Given the lack of correlation between secreted α-defensins1-3 by imMDDC and PVL, we wanted to study whether plasma levels of α-defensins1-3, which are thought to be mainly neutrophil-derived [41], could show some relationship with immunologic and virologic parameters.

**Figure 5:**
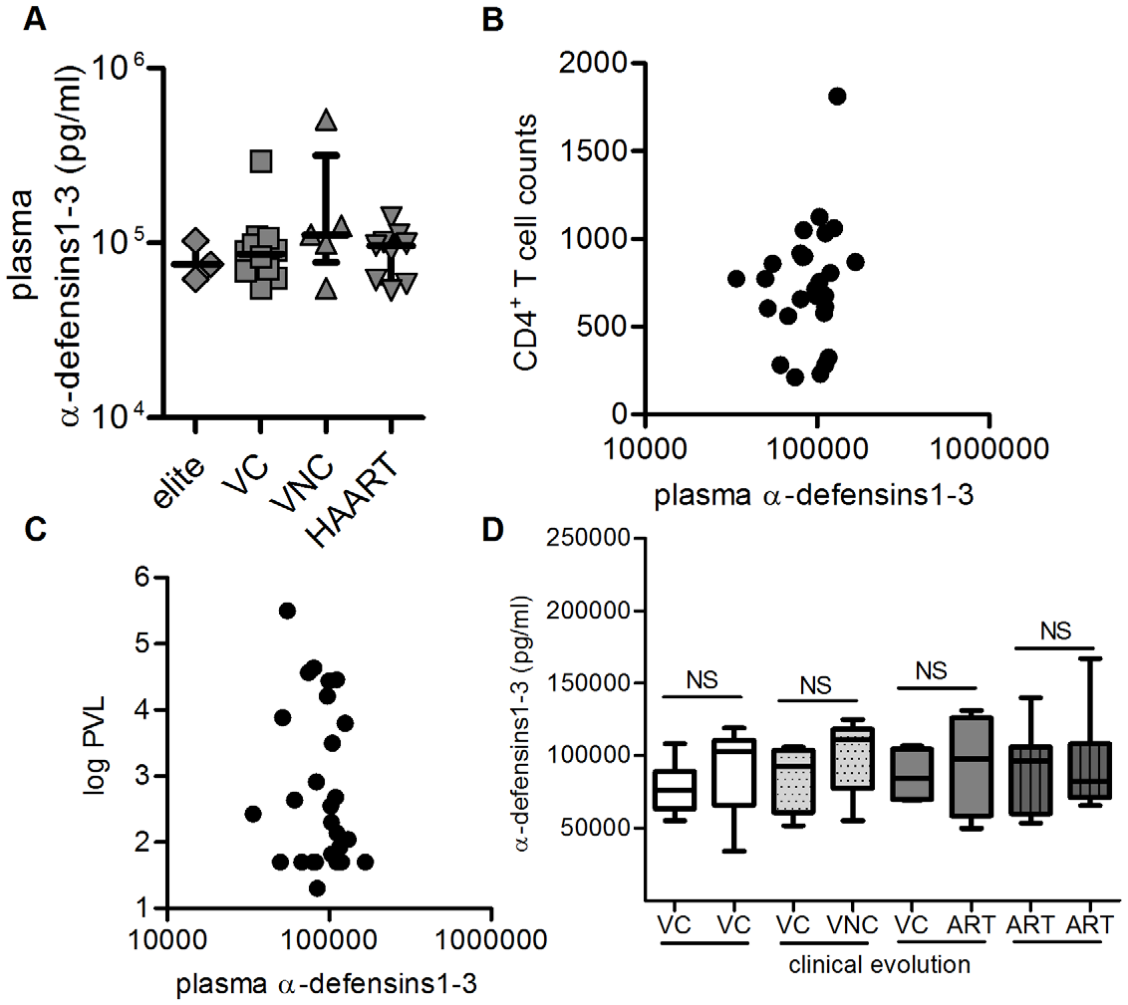
Analysis of plasma levels of α-defensins 1-3. (A) Plasma levels of α-defensins 1-3 detected in the different groups of HIV-infected patients, elite controllers (ELITE; n = 3), viremic controllers (VC; n = 10), viremic non-controllers (VNC; n = 5) and patients receiving HAART (HAART; n = 9). Dots indicate each patient and lines represent median ± interquartile ranges. (B) Correlation between CD4 T cell counts and plasma levels of α-defensins 1-3. (C) Correlation between PVL and plasma levels of α-defensins 1-3. (D) Plasma levels of α-defensins 1-3 in two different determinations over time in patients that remained controllers (VC-VC; white boxes; n = 13), patients that were controllers in the first determination but lost viremic control in the second one (VC-VNC; grey pointed boxes; n = 5), controller patients in the first determination with disease progression and HAART in the second one (VC-ART; grey boxes; n = 8) and patients receiving HAART during both determinations (ART-ART; grey lined boxes; n = 9). Boxes represent interquartile ranges, horizontal lines inside each box indicate the median and whiskers indicate maximum and minimum values. Mann-Whitney test was applied for the statistical analysis of every pair and the differences were not significant (NS).

The plasma levels of α-defensins1-3 were determined using frozen plasma from 35 patients. No significant differences between the different groups of HIV-1-infected patients were found in the plasma levels of α-defensins1-3 (Fig. 5A). It is worth noting the different profile between plasma α-defensins1-3 levels and the levels secreted in the supernatant by imMDDC (Fig. 2C). Furthermore, the analysis of the possible relationship between plasma levels of α-defensins1-3 and CD4 T cell counts or PVL lacked any correlation (Fig. 5B and Fig 5C), even after eliminating the patients with HAART or separating controllers from non-controllers for the analysis (data not shown).

To test the possible influence of the evolution of the disease in the levels of α-defensins1-3 in plasma, two samples separated in time were analyzed for every patient. The results of this analysis are shown in Fig. 5D. No significant differences were observed between plasma levels of α-defensins 1-3 in the two different determinations in patients that remained controllers (Fig. 5D, white boxes). Similarly, no significant differences were found in those patients that were controllers in the first determination but lost viremic control in the second one (Fig. 5D, grey pointed boxes), controller patients in the first determination with disease progression and receiving HAART in the second one (Fig. 5D; grey boxes), and patients receiving HAART during both determinations (Fig. 5D; grey lined boxes).

These data indicate that the differences found in the levels of α-defensins1-3 secreted by imMDDC were independent of plasma levels of these molecules.

### Higher Secretion of α-Defensins1-3 by imMDDC from HIV-Infected Patients Was Associated with Slower Disease Progression

**Figure 6:**
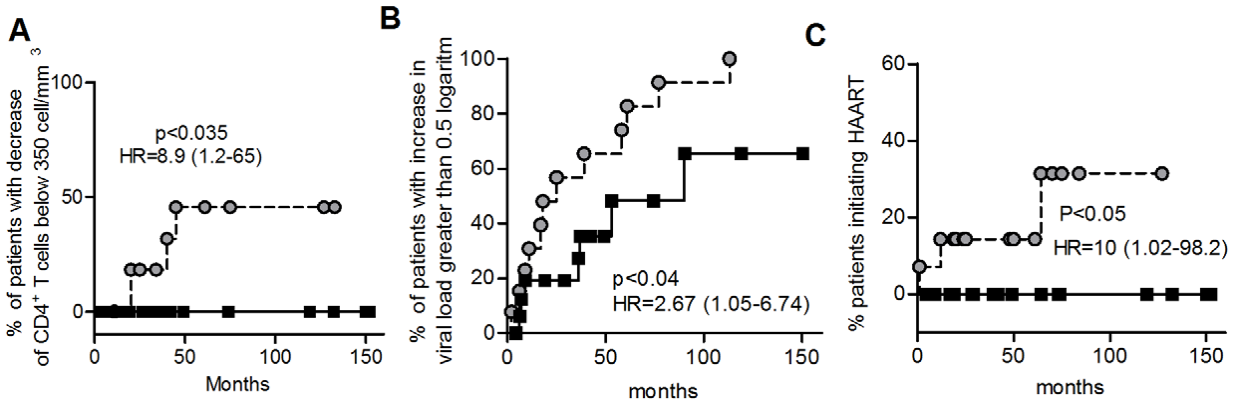
Levels of α-defensins 1-3 secreted by imMDDC were associated with indicators of disease progression. Patients without treatment at the moment of blood extraction for the differentiation of MDDC were divided in two groups according to their secreted levels of α-defensins 1-3 by imMDDC. Levels higher than the median (650 pg/ml) of the whole group of patients (black squares; n = 18) and secreted levels lower than the median (grey circles; n = 12). (A) Percentage of patients with a decrease in the number of CD4 T cells below 350 cel/mm^3^ during follow up time (months). (B) Percentage of patients with an increase in PVL greater than 0.5 logarithms and (C) percentage of patients without HAART at the moment of determination of α-defensins 1-3 levels but started treatment later during the follow up. P values and hazard ratio (HR) with 95% confidence interval values are represented in the figure. Log-Rank test was used to determine statistical differences between curves.

To analyze the possible association of secreted levels of α-defensins1-3 by imMDDC from HIV-infected patients with the risk of disease progression, the untreated patients were separated in two groups: those with levels of secretion over the median of the whole group (650 pg/ml) (hereafter “high secretors”) and those with levels below this median value (hereafter “low secretors”). The median was utilized instead of the mean because it is less influenced by extreme values. Decrease in the number of CD4 T cells, increase in the PVL, and initiation of treatment were considered as indicators of disease progression and were analyzed over time by applying the log-rank test. The percent of patients with a decrease in the number of CD4 T cells below 350 counts over time was analyzed. This decrease in the number of CD4 T cells constitute a critical point to consider treatment according to current guidelines [42], [43]. As shown in fig. 6A, 45% of low α-defensins1-3-secretor patients suffered this CD4 T-cell decrease, while it did not occur in the high α-defensins1-3-secretor patients (p<0.035;HR = 8.9).

In addition, an increase in PVL greater than 0.5 log occurred in all low α-defensins1-3-secretor patients, versus 65% of high secretors (fig. 6B; p<0.04; HR = 2.67). Furthermore, 31% of low secretor patients without treatment at the beginning of the study initiated HAART during the follow-up, while none of the high α-defensins1-3-secretors initiated treatment (fig. 6C; p<0.05; HR = 10).

Taken together these data indicate that those individuals with higher secretion of α-defensins1-3 by imMDDC had a lower risk of disease progression.

## Discussion

In this study we have investigated the production of α-defensins1-3 by immature DC of HIV-infected patients and their possible impact on the disease progression rate. As far as we know, no other studies have approached this issue. Our results revealed that immature DC from HIV-infected patients produced higher levels of α-defensins1-3 than the non-infected control group and these levels were associated with a better control of HIV infection and slower disease progression.

Although the anti-HIV activity of α-defensins1-3 has been clearly demonstrated *in vitro* [5], [8]–[10], [15], [44], their possible protective role during HIV infection *in vivo* remains uncertain. Very few studies[5], [45]–[50] have analyzed α-defensins1-3 at different anatomical sites in low risk healthy controls and HIV-infected subjects to try to determine the physiological and pathological levels of these molecules. For our study we used MDDC, a valid model of *in vivo* myeloid DC[20], [22], [23]. Due to their mucosal localization, myeloid DC *in vivo* are thought to be one of the first cells that encounter the HIV and, after migration to the lymph nodes, would mediate the transmission of HIV-1 virions to CD4 T cells, the main source of HIV-1 replication and dissemination [30]. These *in vivo* myeloid DC might be one of the key cells involved in early HIV infection, and therefore their capacity to produce and release α-defensins1-3 by DC may have physiological relevance.

Our HIV-infected patients included controllers and non-controllers to study the behavior of α-defensins1-3 secretion during opposite situations. The study of controller patients, especially elite controllers, is of particular relevance since these individuals demonstrate that natural control of HIV replication in the absence of antiretroviral therapy is possible[51]. Therefore, the characterization of the protective factors that contribute to this controller state will provide valuable information for new therapeutic approaches.

We found that immature DC from individuals that spontaneously control the infection (i.e. elite controllers and “viremic” controllers) produced higher levels of α-defensins1-3 than the non-controller group (i.e. viremic and treated patients). This higher production occurred in a constitutive manner, in the absence of any *in vitro* stimulation, a characteristic of innate immune mechanisms[52], [53]. Patients with high viremia and patients with undetectable viremia due to antiretroviral therapy produced similar levels of α-defensins1-3, indicating that low PVL alone was not responsible for the increased levels of secreted α-defensins1-3 in controllers. It is worth noting that all selected patients were matched to have conserved levels of CD4 T cells, so the differences observed were not attributable to distinct degrees of immunodeficiency.

We also found that secreted levels of α-defensins1-3 by imMDDC positively correlated with CD4 T cell counts, a parameter of disease progression. However, this correlation was only found in untreated patients and especially in the controller group, but not in non-controllers, even though all selected patients had similar CD4 T cell counts, providing evidence of a relationship between higher α-defensins1-3 production by DC and better immunological state. Interestingly, no correlation was found between PVL and secreted α-defensins1-3 by MDDC. In agreement with our results, in other studies no correlation was found between PVL and α-defensins1-3 expression in lymph nodes[48] or PVL and levels of α-defensins1-3 in breast milk[46].

It should be noted that the analysis of plasma levels of α-defensins1-3 did not show differences between groups of patients. High levels of plasma α-defensins1-3 in humans have only been found during acute infectious processes, such as sepsis, bacterial meningitis or intrauterine infections [54]. Furthermore, we did not observe any correlation between plasma levels of α-defensins1-3 and CD4 T cell counts or PVL. This is not contradictory with our results with MDDC since α-defensins1-3 detected in plasma are mainly secreted by neutrophils[41]. The possible association between plasma levels of α-defensins1-3 and neutrophil activation was not analyzed in our study, but it has been previously found in pregnant and post-partum women [55]. Furthermore, Baroncelli *et al* [45], reported a marked increased in α-defensins1-3 plasma levels only two weeks after SIV post-infection of macaques, which coincided with the peak of viral replication, but following the acute phase of infection the levels of α-defensins1-3 decreased. Therefore, it would be conceivable that no differences were found in the plasma levels of α-defensins1-3 in our chronically-infected patients.

The results found in the analysis of disease progression markers over time further support that a higher production of α-defensins1-3 by immature DC acts as a protective host factor against disease progression. Indeed, regardless their clinical classification, HIV-1-infected patients with higher α-defensins1-3 secretion by immature MDDC showed a delayed disease progression, measured as no decrease in the number of CD4+ T-cells below 350 cell/mm^3^, lower increase in PVL and no initiation of treatment. The association with a decrease in CD4 T cells below 350 cell/mm^3^ is especially relevant since this CD4 T-cell count constitutes a critical point to decide the starting of treatment[42], [43]. The possibility of a genetic predisposition for a higher production of α-defensins1-3 deserves further studies. In fact, the genes encoding for α-defensins1-3 map in a cluster on chromosome 8p23.1 and have been demonstrated to vary in their copy number between individuals[56]–[59]. In this line, recent studies reported higher copy number of the β-defensins HBD-2 and HBD-3 mRNA in oral mucosa from exposed seronegative (ESN) than healthy controls [60] and lower copy number of the gene DEFB104 in HIV-infected than ESN children[61].

*In vivo*, different immature myeloid DC subsets, such as Langerhans cells and interstitial DC, are found positioned in mucosal territories where the natural HIV-1 infection and dissemination occurs. The production of α-defensins1-3 by these *in vivo* myeloid DC subsets has not been investigated, an issue that deserves further investigation. Even though the levels of α-defensins1-3 secreted by imMDDC did not reach the described concentration for direct inactivation of the virus [8], there is the possibility that the amount of α-defensins1-3 produced by these *in vivo* myeloid DC subsets is higher than that produced by our imMDDC. In this regard, it is worth noting that we have recently compared directly isolated circulating myeloid DC (CD1c+ CD19-) and *in vitro* generated imMDDC of the same donors for secretion of α-defensins1-3, and we found that the former secreted a 10-fold higher amount then the latter (M Escribese et al, manuscript in preparation). On the other hand, it is also plausible that *in vivo* the virus would be internalized by immature DC and be localized in intracellular compartments where the concentration of defensins might be locally higher than the levels secreted by imMDDC. Moreover, the reported concentration necessary for direct inactivation of the virus [8] was determined *in vitro* using CD4 T cells for the assays. As it is well known, compared to CD4 T cells, immature myeloid DCs are poorly permissive to HIV-1- replication and therefore it is likely that the concentration of α-defensins1-3 necessary to inhibit the productive infection of DCs is lower than the concentration previously reported to be required using CD4 T cells.

It would be conceivable that a high production of α-defensins1-3 by immature DC could act by damaging the virus prior or after its internalization. This would favor a more efficient viral processing and presentation to CD4+ T cells with a minor rate of infectious HIV transmission. In addition, α-defensins1-3 have important immunoregulatory properties[2], [12], [13], [15], [18], that might modulate HIV replication and immune cell responses in a complex manner, influencing the natural history of HIV infection. Further studies with larger cohorts of HIV-1-infected patients are required to confirm our observations.

In conclusion, we demonstrate that DC from HIV-infected patients that spontaneously control the infection produced higher levels of α-defensins1-3, which positively correlated with CD4 T cell counts and were associated with slower progression. Our results open a new line of investigation and future studies will be needed to determine the possible value of α-defensins1-3 as a diagnostic or therapeutic tool.
